# Dissociative disorder following preeclampsia: A case report

**DOI:** 10.1192/j.eurpsy.2021.1726

**Published:** 2021-08-13

**Authors:** A. Giménez Palomo, M.L. Imaz, I. Pacchiarotti, A. Roca, E. Solé, L. Garcia-Esteve

**Affiliations:** 1 Unit Of Perinatal Mental Health, Hospital Clinic Barcelona, Barcelona, Spain; 2 Department Of Psychiatry And Psychology, University of Barcelona, Barcelona, Spain

**Keywords:** preeclampsia, Perinatal Mental Health, dissociative disorder, hypertension

## Abstract

**Introduction:**

Preeclampsia is a new-onset hypertension with new-onset proteinuria after 20 weeks gestation. Scarce evidence regarding psychiatric effects of preeclampsia is available.

**Objectives:**

To describe a case of a pregnant 24 year-old patient with a premature cesarean section in context of severe preeclampsia and dissociative symptoms.

**Methods:**

Patient referred to a third-level hospital for cesarean section due to a severe preeclampsia at week 32, in whom magnesium sulfate, labetalol perfusion and betamethasone are started. In the puerperium period only labetalol up to 300 mg/6h is maintained.

**Results:**

Due to the appearance of pulsating headache and photophobia, a computerized tomography is conducted, showing bilateral insular and occipital hypodensity related to vasogenic edema. High blood pressure is maintained (177/121 mmHg) despite antihypertensive treatment. A magnetic resonance imaging and an ophthalmologic exam do not show significant abnormalities and blood pressure is stabilized with treatment. However, the patient refers new-onset auditory imperative hallucinations and suicide thoughts, being referred to our Acute Psychiatric Ward for clinical assessment and intervention. Treatment with risperidone 2 mg is started. The day after her admission, she does not refer psychotic symptoms, explaining depersonalization symptoms in the previous 5 days, seeing herself having to choose a knife to commit suicide. After discharge, she maintains reiterative dreams in which she falls down from a building, not presenting dissociative symptoms during the day.

**Conclusions:**

Further evidence regarding psychiatric effects of preeclampsia is needed in order to study the consequences of edema and pharmacological treatment. Blood pressure and psychiatric symptoms monitoring after preeclampsia should also be considered.

**Disclosure:**

No significant relationships.

